# Commensal *Neisseria cinerea* impairs *Neisseria meningitidis* microcolony development and reduces pathogen colonisation of epithelial cells

**DOI:** 10.1371/journal.ppat.1008372

**Published:** 2020-03-24

**Authors:** Rafael Custodio, Errin Johnson, Guangyu Liu, Christoph M. Tang, Rachel M. Exley

**Affiliations:** Sir William Dunn School of Pathology, University of Oxford, Oxford, United Kingdom; INSERM, FRANCE

## Abstract

It is increasingly being recognised that the interplay between commensal and pathogenic bacteria can dictate the outcome of infection. Consequently, there is a need to understand how commensals interact with their human host and influence pathogen behaviour at epithelial surfaces. *Neisseria meningitidis*, a leading cause of sepsis and meningitis, exclusively colonises the human nasopharynx and shares this niche with several other *Neisseria* species, including the commensal *Neisseria cinerea*. Here, we demonstrate that during adhesion to human epithelial cells *N*. *cinerea* co-localises with molecules that are also recruited by the meningococcus, and show that, similar to *N*. *meningitidis*, *N*. *cinerea* forms dynamic microcolonies on the cell surface in a Type four pilus (Tfp) dependent manner. Finally, we demonstrate that *N*. *cinerea* colocalises with *N*. *meningitidis* on the epithelial cell surface, limits the size and motility of meningococcal microcolonies, and impairs the effective colonisation of epithelial cells by the pathogen. Our data establish that commensal *Neisseria* can mimic and affect the behaviour of a pathogen on epithelial cell surfaces.

## Author summary

Commensal and pathogenic bacteria can establish long term relationships with their hosts. Despite this, very little is known about the processes of attachment, replication and organisation of commensal bacteria at epithelial surfaces. In this work, we have examined how *Neisseria cinerea*, a typically commensal species that colonises the human nasopharynx similar to the closely-related pathogen *Neisseria meningitidis*, engages with human epithelial cells. We show that *N*. *cinerea* on human epithelial cells mimics some of the behaviour of the meningococcus, but have identified subtle differences that distinguish the two species. Furthermore, we show that the presence of *N*. *cinerea* affects the interaction of *N*. *meningitidis* with epithelial cells, providing evidence that the interaction between two closely related species can affect pathogen colonisation of the epithelial surface.

## Introduction

*Neisseria meningitidis* is an important cause of septicaemia and meningitis [1]. Despite being a deadly pathogen, acquisition of *N*. *meningitidis* most often results in asymptomatic colonisation of the nasopharynx. *N*. *meningitidis* is carried by approximately 10–40% of the human population [2] and this niche therefore provides a reservoir for person-to-person transmission, and is the initial barrier to invasive disease [1]. Adhesion to epithelial cells is key for colonisation and is mediated largely by type IV pili (Tfp) [3, 4] which induce localisation of host proteins such as CD44 at the site of meningococcal attachment [5, 6], while ezrin, actin and cholesterol accumulate beneath adherent bacteria [6–9]. Meningococci also induce extensions of the plasma membrane of epithelial cells [9–11] and form microcolonies on the surface, which fuse, expand and disperse, allowing dissemination of bacteria [12, 13].

Within the nasopharynx the meningococcus exists with a community of microorganisms. This local microbiota includes several other *Neisseria* species [14, 15] which are generally considered to be ‘commensal *Neisseria*’ although several of these species can also occasionally cause disease [16–18]. Growing evidence suggests that the microbiota can impact host-pathogen interactions and plays an important role in preventing pathogen expansion [19, 20]. In the case of *Neisseria* species, human challenge studies have shown that nasal inoculation with *Neisseria lactamica* can reduce meningococcal carriage and acquisition [21] although the molecular mechanisms underpinning such observations remain unknown. Therefore, further understanding of the interaction of pathogenic and commensal *Neisseria* with the host and with each other is needed.

*Neisseria cinerea* is considered as a commensal *Neisseria* species [16, 18, 22] and has been identified as a member of the human oral and nasal microbiota [23]. Data on carriage of this organism are relatively limited however, it has been independently isolated from the nasopharynx of adults [22] and children [24]. *N*. *cinerea* is closely related to the meningococcus and harbours genes involved in virulence [25–27]. We have previously shown that *N*. *cinerea* adheres to epithelial cells, forms microcolonies, and closely associates with microvillus-like structures, similar to those observed during meningococcal adhesion. However, unlike the meningococcus *N*. *cinerea* does not require Tfp for attachment and their role during *N*. *cinerea* colonisation is not known [28].

Here, we characterised the interactions of *N*. *cinerea* with human epithelial cells and examined the impact of this bacterium on the behaviour of *N*. *meningitidis* at the cell surface. We demonstrate that *N*. *cinerea* uses similar molecules to *N*. *meningitidis* during colonisation, but define features that distinguish pathogen-host and commensal-host interactions. Importantly, we demonstrate that the presence of *N*. *cinerea* reduces meningococcal association with cells and show that *N*. *cinerea* can limit the motility and size of meningococcal microcolonies. Taken together, our data highlight that the presence of a related commensal can influence pathogen behaviour during interactions with human respiratory epithelial cells.

## Results

### *N*. *cinerea* co-localises with components of cortical plaques on epithelial cells

**Fig 1:**
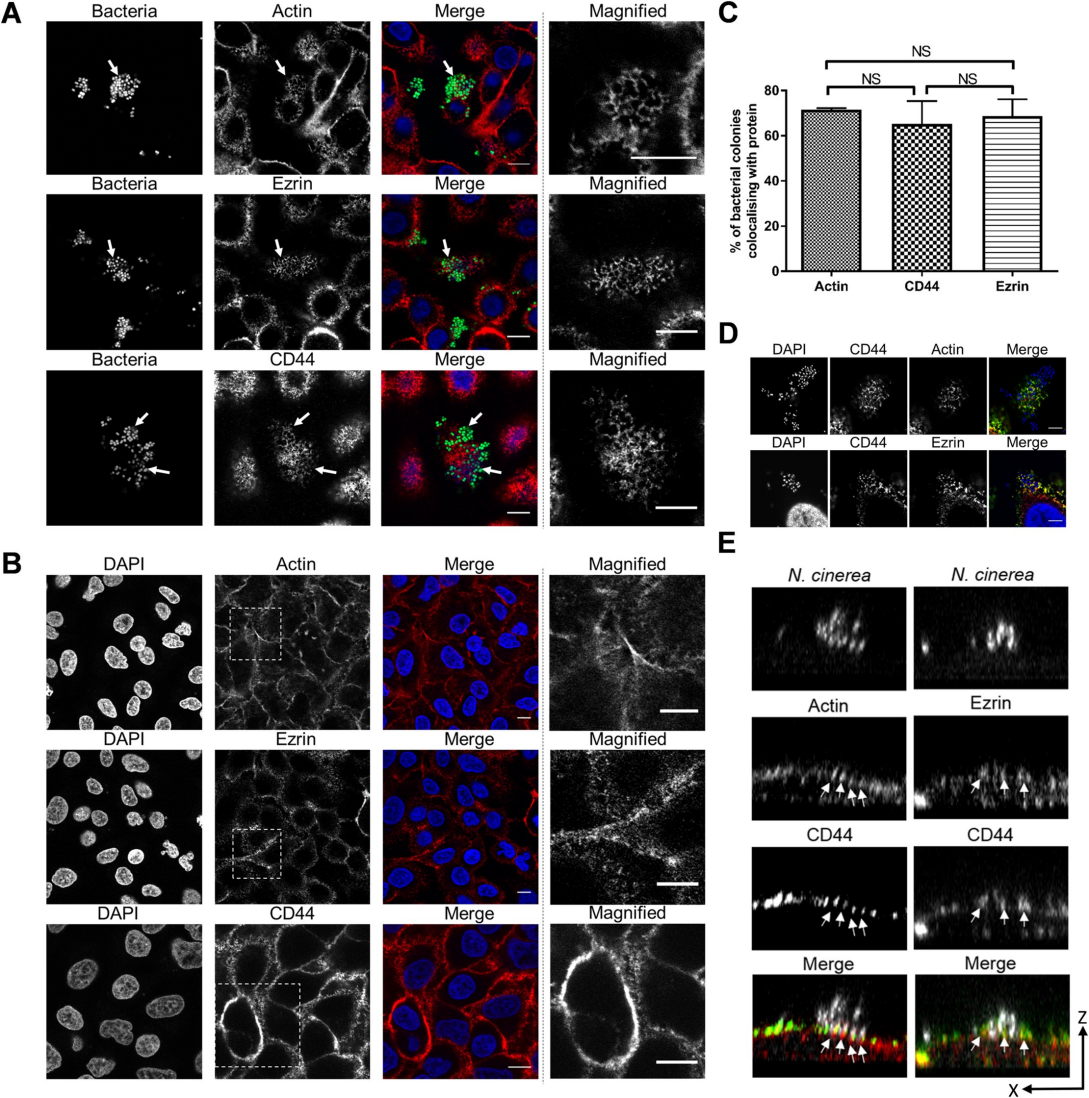
*N*. *cinerea* co-localises with components of cortical plaques on epithelial cells. (**A**) Epithelial cells were infected for 3 h with *N*. *cinerea* expressing GFP and stained for CD44, ezrin or actin. Bacteria co-localised with each protein (white arrows); magnified areas in the panels on the right show the honeycomb-like arrangement of each protein. (**B**) Non-infected A549 cells were immunostained for CD44, ezrin or actin and analysed by microscopy. Magnified areas shown in the panels on the right do not show a honeycomb-like arrangement. Scale bars, 10 μm. (**C**) Frequency of co-localisation of each protein in honeycomb-like arrangement at the site of attachment was determined by scoring 50 microcolonies. Data shown represent the mean ± SD of three independent experiments; NS, not significant. (**D**) Epithelial cells were infected for 3 h with *N*. *cinerea* and double fluorescence labelling was performed. Actin (red) and CD44 (green) in the top panels; or ezrin (red) and CD44 (green) in the bottom panels. Scale bars correspond to 10 μm. (**E**) XZ sections of cells dual labelled for actin (red) and CD44 (green), or CD44 (green) and ezrin (red). Bacteria and nuclei were stained with DAPI (white). Arrows indicate cellular protrusions enriched with actin-CD44 or ezrin-CD44.

Upon interaction with epithelial cells, the meningococcus induces cortical plaque formation, which involves recruitment and rearrangement of cell components into honeycomb-like structures [6, 8]. Therefore, we first sought to determine whether molecules involved in cortical plaque formation are recruited to sites of *N*. *cinerea* attachment. We infected confluent monolayers of A549 cells with *N*. *cinerea* expressing GFP for 3 h, and examined the distribution of actin, ezrin and CD44 by immunofluorescence microscopy. All three components localised underneath *N*. *cinerea* microcolonies (**Fig 1A**). Although actin, ezrin or CD44 were not as dramatically condensed at the attachment site as during meningococcal adhesion to A549 cells (S1 **Fig**), honeycomb-like structures were detected which were not observed in uninfected cells (**Fig 1B**). At 3 h post infection (hpi), over 60% of *N*. *cinerea* microcolonies co-localised with actin, CD44 or ezrin (**Fig 1C**). Dual labelling demonstrated that CD44 localised together with F-actin and ezrin (**Fig 1D**) and XZ optical sections revealed that actin-CD44 or ezrin-CD44 were detected at the tips of cellular protrusions associated with bacteria (**Fig 1E**). Therefore these data suggest that commensal and pathogenic *Neisseria* exploit similar host proteins during interactions with epithelial cells.

**Fig 2:**
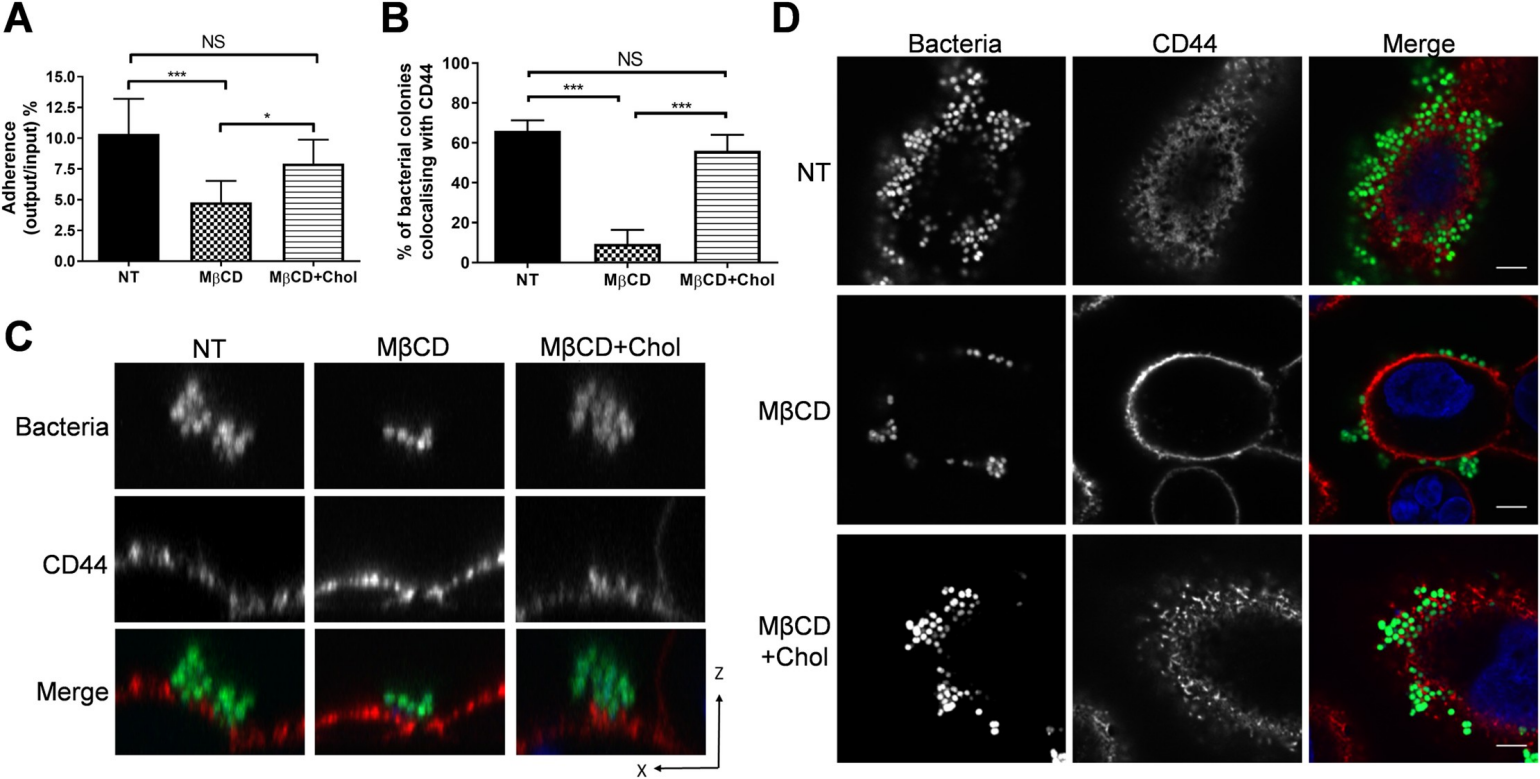
*N*. *cinerea* requires a cholesterol-rich plasma membrane for an efficient host-cell colonisation. (**A**) Epithelial cells were treated with MβCD or MβCD and cholesterol (MβCD+Chol) then infected for 3 h. Adhesion of *N*. *cinerea* was reduced in the presence of MβCD and restored to wild-type levels with addition of cholesterol. (**B**) Frequency of CD44 co-localisation in honeycomb-like structures with adherent *N. cinerea* in the presence of MβCD or MβCD+Chol was compared to non-treated cells (NT). (**C**) XZ-section images of microcolonies attached to host cells were acquired by confocal microscopy. CD44 was stained with anti-CD44 (red) and cell-associated bacteria are in green. (**D**) CD44 localisation (red) in cells treated with MβCD or MβCD+Chol and infected for 3 h with *N*. *cinerea* expressing GFP (green) at an MOI of 100. DNA was stained with DAPI (shown blue in merge). Scale bar, 10 μm. Data shown are the mean ± SD of three independent experiments: NS, not significant; *p <0.05; ***p<0.0005 using one-way ANOVA test for multiple comparison.

We also investigated the contribution of cholesterol to *N*. *cinerea* adhesion as it is recruited to the site of meningococcal attachment [10]. Treatment of cells with the cholesterol depleting agent MβCD reduced *N*. *cinerea* adhesion by 50%; this was reversed by the addition of exogenous cholesterol (**Fig 2A**). However, there was no enrichment of cholesterol underneath *N*. *cinerea* microcolonies (S1 **Fig**), although cholesterol depletion reduced CD44 colocalisation with bacteria (**Fig 2B**), and resulted in a single layer of bacteria on the cell surface rather than multi-layered aggregates observed on cholesterol replete cells (**Fig 2C and 2D**). These findings indicate that like *N*. *meningitidis*, *N*. *cinerea* requires a cholesterol-rich plasma membrane to effectively adhere to cells but does not actively recruit cholesterol to the attachment site.

**Fig 3:**
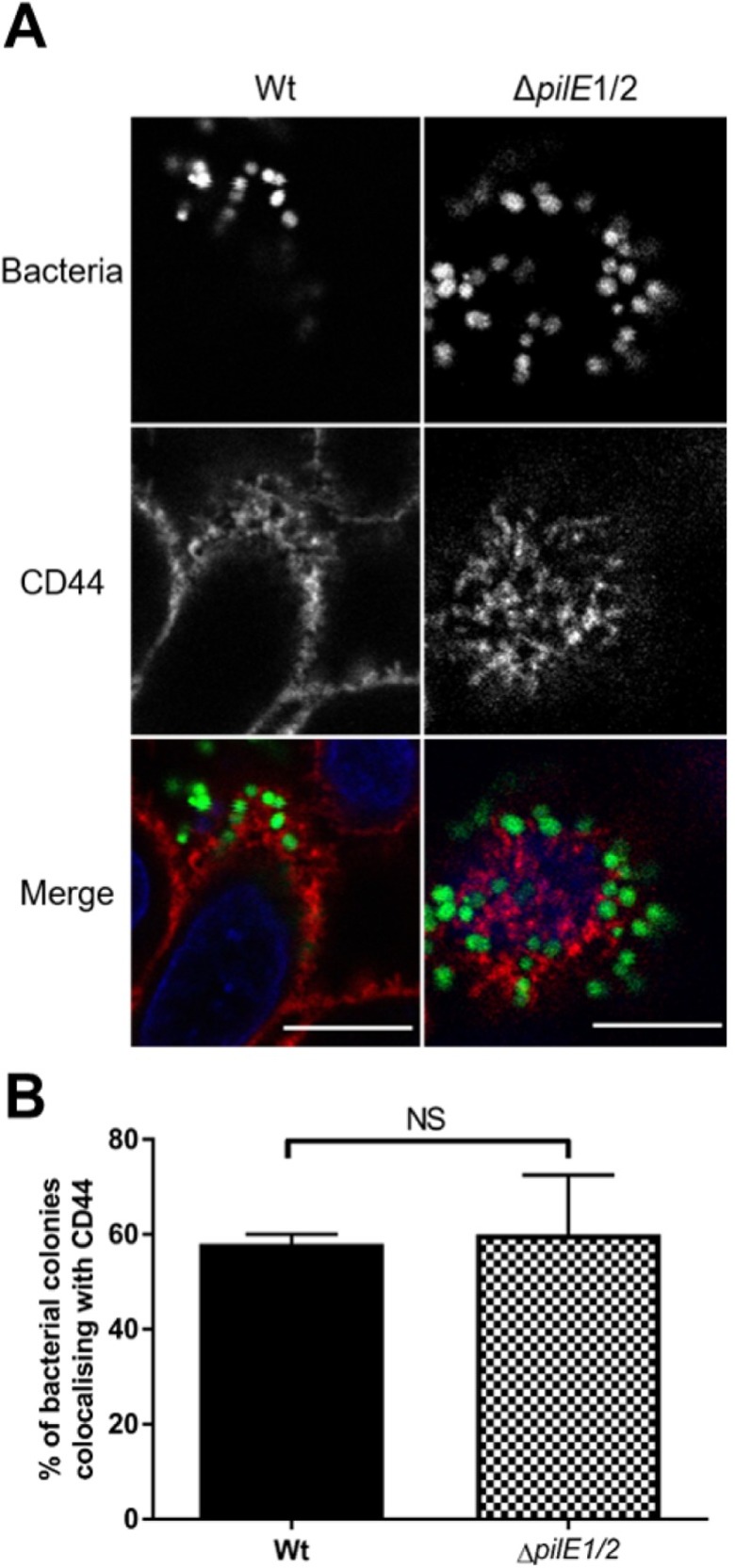
Tfp are not required for colocalisation of *N*. *cinerea* with CD44 honeycomb-like structures. (**A**) Epithelial cells were infected with wild-type *N*. *cinerea* 346T (Wt) or *N*. *cinerea* 346TΔ*pilE*1/2 both expressing GFP (green in merge) at an MOI of 100. At 3 hpi, cells were stained for CD44 (red in merge). Scale bar, 10 μm. (**B**) Quantification of CD44 colocalisation with bacterial colonies. Results represent the mean ± SD of three independent experiments. NS, not significant using unpaired two-tailed Student’s t-test.

Finally, we determined whether Tfp are required for the formation of honeycomb-like structures observed during *N*. *cinerea* adhesion. *N*. *meningitidis* Tfp are essential for efficient adhesion [4] and for signalling that leads to cortical plaque formation [29], but *N*. *cinerea* Tfp are dispensable for efficient adhesion [28]. Epithelial cells were infected with a Tfp-deficient mutant (346TΔ*pilE*1/2) and labelled with α-CD44 pAbs at 3 hpi. Interestingly there was no significant difference in CD44 localisation below microcolonies of the wild-type and the *pilE*1/2 mutant (58% *vs*. 60%, **Fig 3**). Thus rearrangements of the cortical cytoskeleton observed upon adhesion of *N*. *cinerea* are likely to occur *via* mechanisms that are distinct from those described for *N*. *meningitidis*.

### Dynamics of *N*. *cinerea* adhesion to epithelial cells

**Fig 4:**
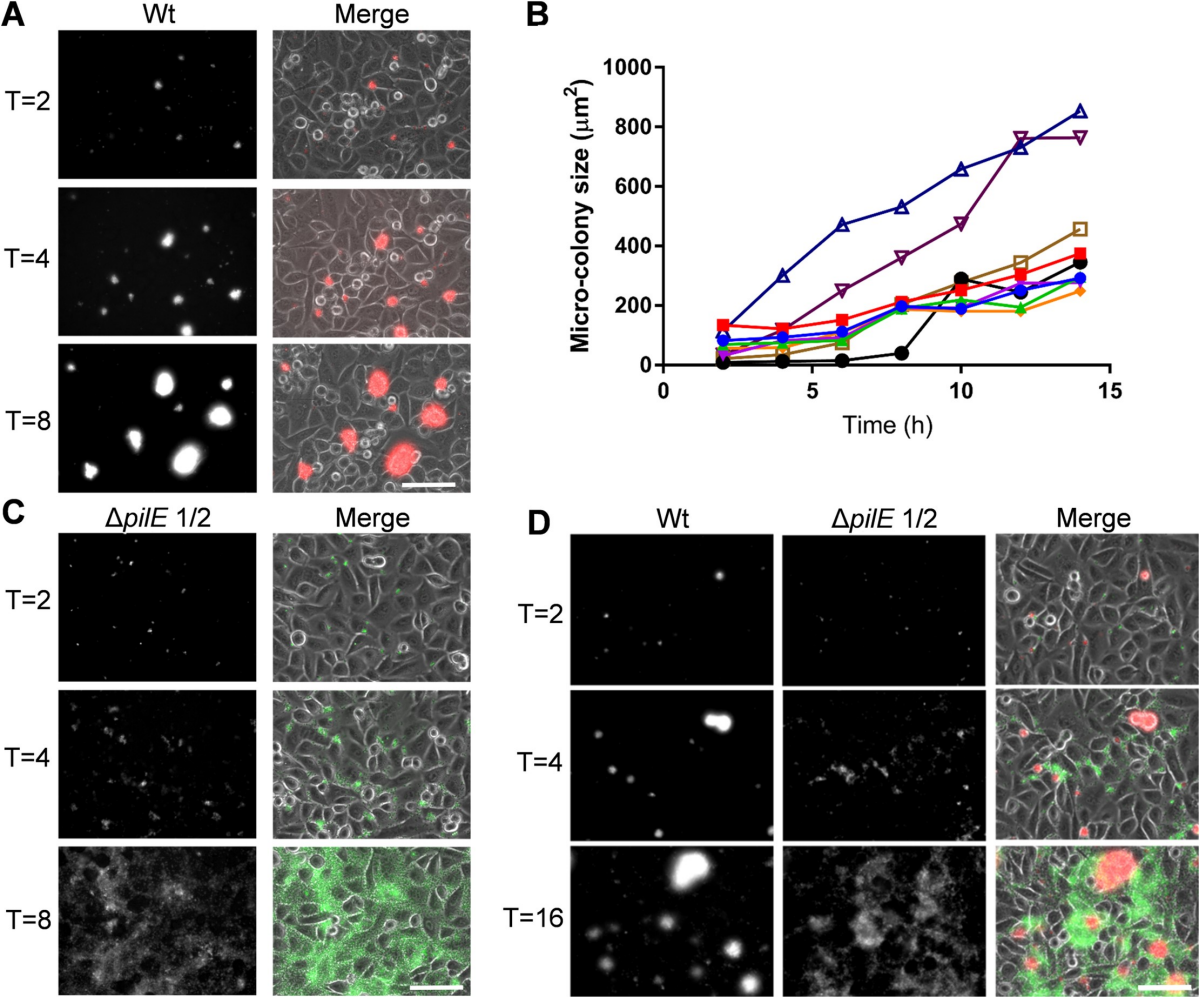
*N*. *cinerea* Tfp are required for microcolony formation and fusion. **(A)** Epithelial cells were infected with *N*. *cinerea* wild-type (Wt) expressing sfCherry for 16 h. Images were captured at 10 min intervals; time points 2, 4 and 8 h are shown. (**B**) Microcolony size was quantified by measuring their surface area. Each line corresponds to a single microcolony tracked over time. Data shown are for eight microcolonies from one representative experiment of three independent experiments. (**C**) Epithelial cells infected with *N*. *cinerea* 346TΔ*pilE*1/2 expressing GFP. Images were captured at 10 min intervals, and images from 2, 4 and 8 h post infection are shown. (**D**) Epithelial cells co-infected with wild-type *N*. *cinerea* (Wt, red in merge) and the *pilE* mutant (Δ*pilE*1/2, green in merge). Images are representative of three independent experiments performed in triplicate. In each case merged panels show both phase-contrast and fluorescence channels (GFP and Texas Red). Scale bars, 75 μm.

Meningococci proliferate on cells and form microcolonies through Tfp-mediated interactions, enhancing the ability of bacteria to withstand shear forces [30]. Furthermore, on epithelial cells microcolony dispersal is induced by lactate [13] or Tfp modification [12] and may allow bacteria to adhere to new sites, disseminate to new hosts, or invade [12, 13]. To determine whether *N*. *cinerea* shows similar dynamics, we analysed microcolony formation on epithelial cells using live imaging. Cells were infected with *N*. *cinerea* 346T expressing sfCherry or 346TΔ*pilE*1/2 expressing GFP, left for 1.5 h (to allow bacterial adhesion), then washed to remove non-cell associated bacteria, and monitored over 16 h. In cells infected with the wild-type 346T, microcolonies appeared on the cell surface within 2 hpi, increased in size and number over time and often fused to form larger structures, which were initially multi-lobed, but then became spherical (**Fig 4A** and **4B**, **S1 Movie**). Of note, we found no evidence of *N*. *cinerea* microcolony dispersal over the 16 h period. Microcolonies of 346TΔ*pilE*1/2 were smaller than wild-type at 2 hpi, and although the non-piliated mutant adhered to cells and spread over the surface throughout the period of infection, it was mainly as single or small groups of bacteria (*i*.*e*. less than 20 bacteria) (**Fig 4C, S2 Movie**).

To investigate whether non-piliated bacteria could integrate into microcolonies of the wild-type strain, we co-infected epithelial cells with 346T expressing sfCherry and 346TΔ*pilE*1/2 expressing GFP. Bacteria lacking Tfp failed to form mixed microcolonies with wild-type bacteria and remained on the periphery of large assemblies (**Fig 4D, S3 Movie**). Therefore, while Tfp are dispensable for the adhesion of *N*. *cinerea* to epithelial cells [28], similar to *N*. *meningitidis* they play a critical role in dynamic interactions between bacteria on the cell surface, and contribute to the formation and morphology of microcolonies.

### *N*. *cinerea* and *N*. *meningitidis* interact on epithelial cells

**Fig 5:**
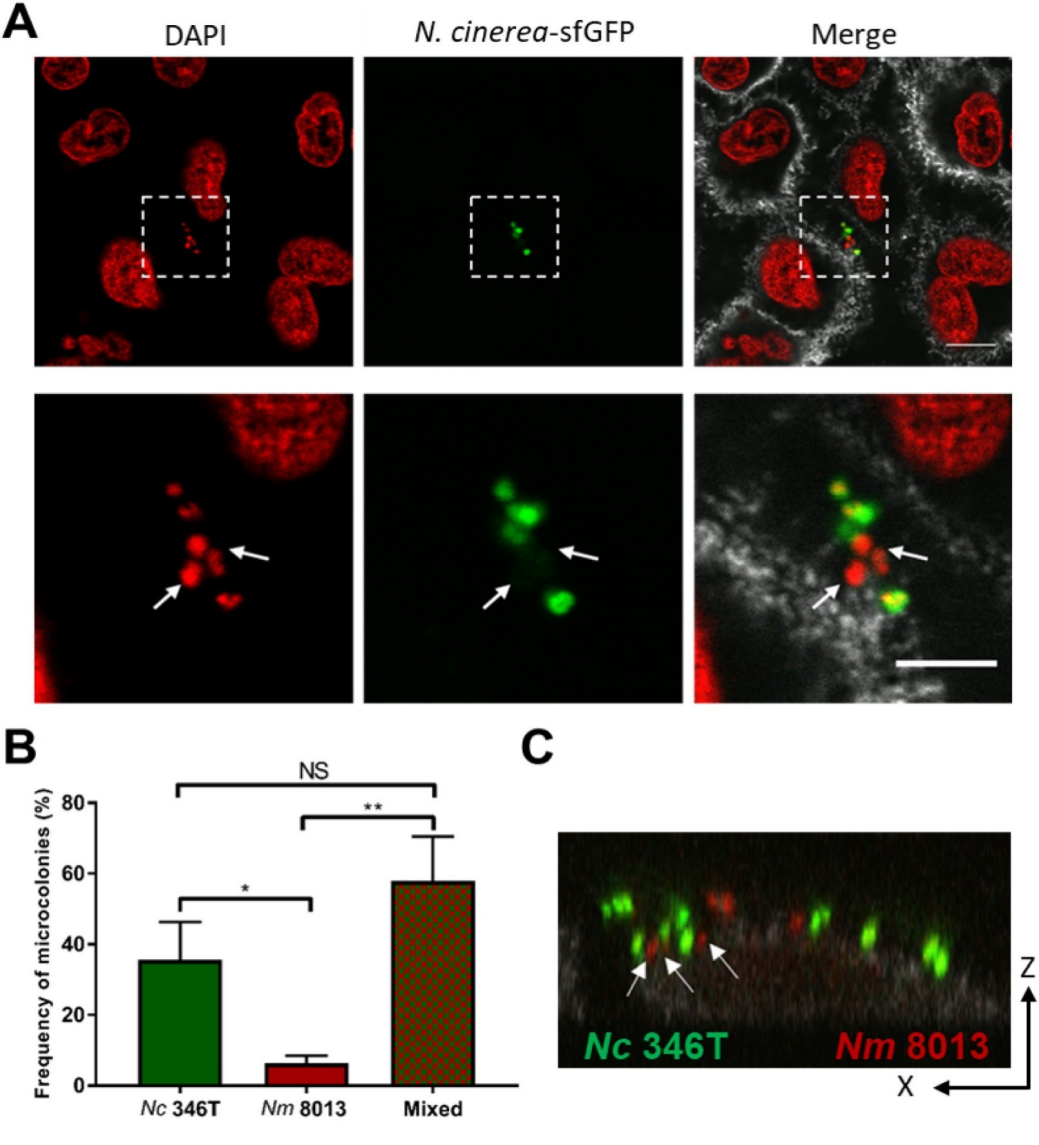
*N*. *cinerea* and *N*. *meningitidis* form mixed microcolonies on epithelial cells. (**A)** Representative images of epithelial cells co-infected with *N*. *cinerea* (*N*. *cinerea*-sfGFP) and *N*. *meningitidis* for 3 h. Lower panels are enlarged images of boxed area in upper panels. Nuclei and bacterial DNA were stained with DAPI (red). Each DAPI stained spot corresponds to a single bacterium (left-hand and merge panels). White arrows indicate *N*. *meningitidis* (DAPI positive, GFP negative) within a mixed microcolony. CD44 is shown in grey (merge panels). Scale bar, 10 μm. (**B**) Frequency of mixed or single species microcolonies quantified by confocal microscopy. Data shown are from at least 150 microcolonies in three independent experiments; the number of mixed microcolonies was expressed as a percentage of all microcolonies. NS, not significant; *, *p*<0.05; **, *p*< 0.005 (one-way ANOVA). (**C**) XZ optical section analysis of a mixed microcolony containing *N*. *meningitidis* (*Nm* 8013, red) and *N*. *cinerea* (*Nc* 346T, green) on epithelial cells. White arrows show *N*. *meningitidis* in close contact with *N*. *cinerea* on the cell surface.

**Fig 6:**
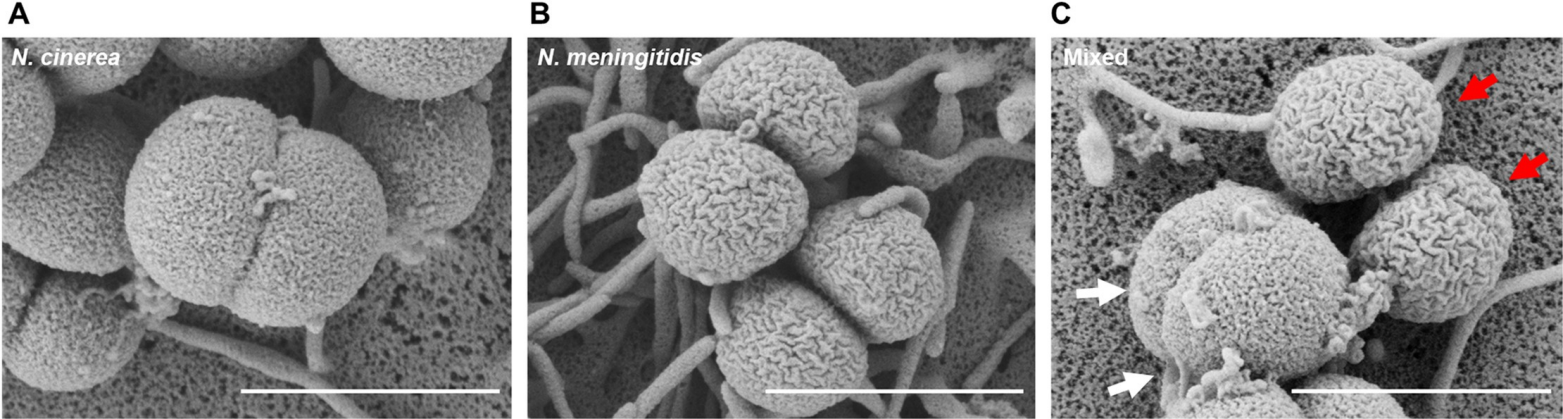
Scanning electron microscopy of mixed *Neisseria* microcolonies on epithelial cells. SEM analysis of A549 cells infected for 6 h with *N*. *cinerea* 346T (**A**), *N*. *meningitidis* 8013 (**B**) or cells co-infected with both species (**C**). White arrows indicate *N*. *cinerea*, and red arrows highlight *N*. *meningitidis*. Scale bars, 1 μm.

Next we analysed whether *N*. *cinerea* and *N*. *meningitidis* colocalise on epithelial cells. Using *N*. *cinerea* expressing GFP and a wild-type, capsule-expressing serogroup C strain of *N*. *meningitidis*, we infected A549 cells with both species at a 1:1 ratio (MOI of 50 for each). After 3 h, more than 50% of microcolonies contained both *N*. *cinerea* and *N*. *meningitidis* (**Fig 5A** and **5B**), with both species in close proximity to the epithelial cell surface within individual microcolonies (**Fig 5C**). We also examined A549 cells infected for 6 h with each species individually or together by scanning electron microscopy (SEM). Observation of cells infected with single species (**Fig 6A** and **6B**) revealed that *N*. *cinerea* have a “coarse-grained” membrane architecture (**Fig 6A)** whereas meningococci have a reticulated surface (**Fig 6B).** These morphological differences allowed us to identify each species during co-infection. As shown in **Fig 6C**, we detected meningococci and *N*. *cinerea* clustered together on the cell surface, confirming that the two species can be found in close proximity and localise together at sites of attachment on epithelial cells.

### *N*. *cinerea* reduces *N*. *meningitidis* association with epithelial cells

**Fig 7:**
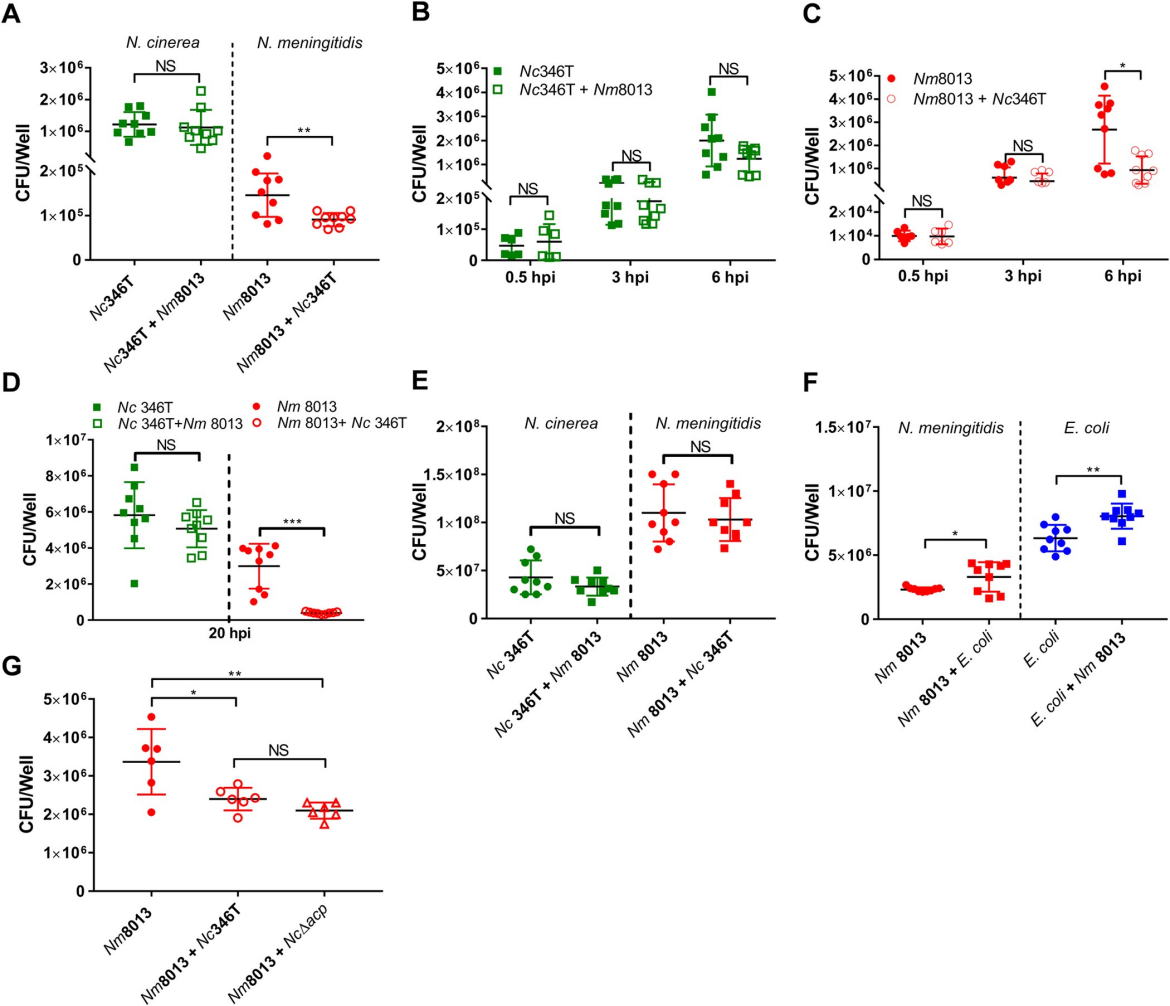
*N*. *cinerea* reduces association of *N*. *meningitidis* with epithelial cells. **(A)** Cells were infected with *N*. *cinerea* (*Nc*346T) for 4.5 h prior to infection with *N*. *meningitidis* (*Nm*8013). The number of cell associated bacteria of each species was determined 1.5 h later. Results are the mean ± SD of three independent experiments carried out in triplicate. NS, not significant; ***p<0.0005 (unpaired two-tailed Student’s t-test). (**B** and **C**) Epithelial cells were infected with *N*. *meningitidis* (*Nm* 8013) alone or with *N*. *cinerea* (*Nc* 346T). The number of cell associated bacteria (CFU/mL) was determined at time points as indicated. Filled shapes show the number of CFU/well in single infections, while empty shapes show the number of CFU/well in co-infections. Each data point represents a single well from three independent experiments conducted in triplicate. NS, not significant; *, *p*<0.05 (unpaired two-tailed Student’s t-test). (**D**) Epithelial cells were infected with *N*. *meningitidis* (*Nm* 8013) alone or co-infected with *N*. *cinerea* (*Nc* 346T) at a ratio of 1:100 (*Nm* 8013 to *Nc* 346T) for 20 h. **(E)** Single and mixed cultures of *N*. *meningitidis* (8013) and *N*. *cinerea* (346T) were grown in the absence of cells for 6 hrs, and the number of bacteria was determined by selective plating. Results are the mean +SD of three independent experiments carried out in triplicate. NS, not significant. **(F)** Epithelial cells were infected with *N*. *meningitidis* (*Nm* 8013) alone or co-infected with *E*. *coli* (BL21 pET21b) at an MOI of 50 for each strain. Cell associated *N*. *meningitidis* and *E*. *coli* (CFU/well) was determined at 6 hpi. Filled circles show *Nm*8013 (red) and *E*. *coli* (blue) in single infections; filled squares show *Nm*8013 (red) and *E*. *coli* (blue) in co-infection. Results are the mean ± SD of 9 replicates from three independent experiments. NS, not significant; *p<0.05; **p<0.005 (unpaired two-tailed Student’s t-test). **(G)** Epithelial cells were infected with *N*. *meningitidis* (*Nm* 8013) alone or co-infected with wild-type *N*. *cinerea* (*Nc*346T) or a mutant lacking ACP (*Nc*Δ*acp*). At 6 hpi, cell associated *N*. *meningitidis* was quantified and presented as CFU per well. Filled circles show *Nm*8013 bacterial numbers in single infections; empty circles or empty triangles show levels of *Nm*8013 in co-infection. Results are the mean ± SD of at least three independent experiments carried out in duplicate. NS, not significant; *, *p*<0.01; **, *p*<0.001 (one-way ANOVA test for multiple comparison).

We next sought to ascertain whether *N*. *cinerea* has any impact on the interaction of *N*. *meningitidis* with epithelial cells. We initially performed assays in which confluent A549 monolayers were pre-infected with *N*. *cinerea* at an MOI of 50 for 4.5 h, then washed and infected with *N*. *meningitidis* at an MOI of 50 for 1.5 h. The number of bacteria of each species associated with monolayers was ascertained by plating to selective media and quantifying CFU. As shown in **Fig 7A**, there was no difference in the number of *N*. *cinerea* recovered from cells irrespective of the presence of *N*. *meningitidis*. However, pre-infection with *N*. *cinerea*, significantly reduced the number of *N*. *meningitidis* associated with cells relative to the number of meningococci associated to cells without *N*. *cinerea* (approximately 40%, *p* = 0.005).

Next we analysed whether this effect was also observed when cells are infected simultaneously with *N*. *cinerea* and *N*. *meningitidis*. Monolayers of A549 cells were infected with either *N*. *cinerea* or *N*. *meningitidis* individually, or with both species in a 1:1 ratio, in each case at an MOI of 50, and bacteria were recovered at 0.5, 3 and 6 hpi. As expected, the number of both species associated with cells increased over time (**Fig 7B** and **7C**). There was no difference in *N*. *cinerea* cell association in the presence or absence of *N*. *meningitidis* (**Fig 7B**). In contrast, by 6 hpi the level of *N*. *meningitidis* associated with cells was reduced by ~ 65% when *N*. *cinerea* was present (*p* = 0.01, **Fig 7C**). Increasing the ratio of *N*. *cinerea* to *N*. *meningitidis* to 100:1 and the length of infection led to a more marked decrease in *N*. *meningitidis* association with cells (~85% reduction relative to single infection, *p*<0.0001), without discernible impact of the meningococcus on *N*. *cinerea* (**Fig 7D**). As controls, we incubated both species together in a 1:1 ratio in tissue culture media in the absence of epithelial cells, and infected A549 cells with *N*. *meningitidis* +/- *Escherichia coli*. Results demonstrate that there was no direct antagonism between *N*. *cinerea* and *N*. *meningitidis* in the absence of cells (**Fig 7E**), and that the presence of *E*. *coli* did not reduce cell-association of *N*. *meningitidis* (**Fig 7F**). Therefore, our data indicate that *N*. *cinerea* specifically reduces meningococcal association with epithelial cells.

One possible explanation for these findings is that the related species compete for binding sites on the cell surface. Tfp are major adhesins for *N*. *meningitidis* [4, 6, 28, 31], but not for *N*. *cinerea* [28]. Therefore we first sought to identify outer membrane adhesins of *N*. *cinerea* that are shared with *N*. *meningitidis*. Several surface proteins are involved in adhesion of meningococci to epithelial cells, including Opacity proteins [32], NHBA [33], NadA [34] and ACP [35]. Previous sequence analysis has shown that *nhba* and *opa* are not present in *N*. *cinerea* [27, 36]. We found that NadA and ACP in *N*. *cinerea* 346T have approximately 50% and 88% amino acid identity respectively to homologues in *N*. *meningitidis*, but adhesion assays revealed that only ACP contributes to *N*. *cinerea* 346T adhesion (S2 **Fig**). We therefore performed co-infection experiments using *N*. *meningitidis* 8013 in the presence of either wild-type *N*. *cinerea* (346T) or 346TΔ*acp*. Wild-type *N*. *cinerea* and Δ*acp* reduced the number of meningococci associated with cells to the same extent (~40% *p* = 0.003, **Fig 7G**), indicating that even in the absence of ACP, *N*. *cinerea* impairs the association of *N*. *meningitidis* with cells. Therefore, competition for an ACP receptor is unlikely to be the underlying mechanism of reduced association of meningococci with epithelial cells.

### Presence of *N*. *cinerea* hinders meningococcal microcolony development and motility

**Fig 8:**
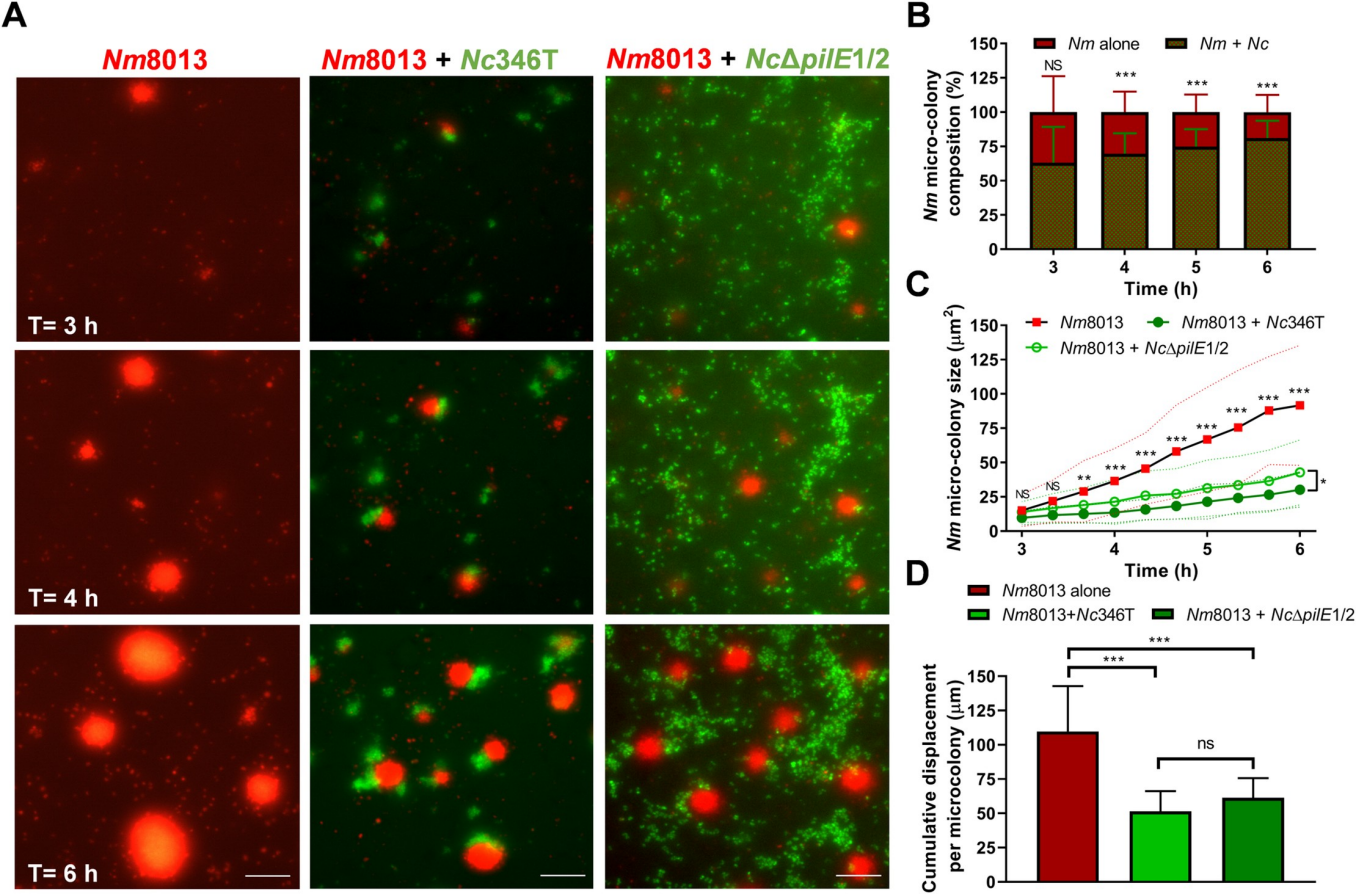
*N*. *cinerea* restricts *N*. *meningitidis* microcolony motility and expansion. **(A)** Representative time-lapse images of epithelial cells infected with *N*. *meningitidis* pNCC101::sfCherry alone (red, *Nm*8013), or with *N*. *cinerea* 346T expressing GFP (green, *Nc*346T) or *N*. *cinerea* 346TΔ*pilE1/2* expressing GFP (green, *Nc*Δ*pilE1/2*). Time points 3, 4 and 6 hpi are shown. Scale bar, 50 μm. (**B**) The frequency of microcolonies containing *Nc*346T and *Nm*8013 assessed by time-lapse microscopy. Most microcolonies harbour both species. Data shows the mean + SD of at least 700 *Nm*8013 microcolonies from three independent experiments. NS, not significant; ***, *p*<0.0005 (unpaired two-tailed Student’s t-test). (**C**) The size of *N*. *meningitidis* aggregates in single and mixed infections was quantified by measuring the area of meningococcal microcolonies. Each point shows the mean ± SD (dotted lines) of 36 microcolonies from three independent experiments. NS, not significant; *, *p*<0.01; **, *p*<0.001; ***, *p*<0.0005 (one-way ANOVA test). (**D**) Total distance travelled by *N*. *meningitidis* microcolonies in presence or absence of *N*. *cinerea*. Cumulative microcolony displacement was calculated based on the total distance covered per microcolony between 3 and 6 hpi. Data shown represent the mean + SD of 36 microcolonies tracked from three independent experiments. NS, not significant; ***, *p*<0.0005 (one-way ANOVA test for multiple comparisons).

Next we used live cell imaging to visualise the interplay of *N*. *cinerea* and *N*. *meningitidis* on epithelial cells and to gain insights into how the presence of the commensal species impacts the pathogen. Monolayers were infected with one or both species at an MOI of 50 and incubated for 6 h. Consistent with previous reports [30, 37, 38] *N*. *meningitidis* formed motile microcolonies which enlarged over time (**Fig 8A**, **S4 Movie**). We observed merging of meningococcal microcolonies and the rapid formation of spherical aggregates, similar to cell sorting through pilus mediated interactions shown previously with *Neisseria gonorrhoeae* [38, 39]. When cells were infected with both *N*. *meningitidis* and *N*. *cinerea* we observed formation of mixed microcolonies. At 6 hpi, 81% of microcolonies contained both species (**Fig 8B**), however, the two species did not form well-mixed aggregates and microcolonies had a bi- or multi-lobed morphology (**Fig 8A**, **S5 Movie**). As expected based on our earlier findings (**Fig 4D**), this was dependent upon the expression of pili, as co-infection with *N*. *cinerea* Δ*pilE*1/2 did not result in the formation of mixed species microcolonies (**Fig 8A**).

We noted that the expansion of meningococcal microcolonies was significantly reduced by the presence of *N*. *cinerea*; for example at 6 hpi in single infection meningococcal microcolonies reached an average cross sectional area of 95 μm^2^ (SD, ±44) whereas in mixed infection meningococci in mixed microcolonies had an average cross sectional area of 30 μm^2^ (SD, ±13, **Fig 8C**, *p*<0.0001). Interestingly, a similar effect was observed with *N*. *cinerea* 346T*pilE*Δ1/2 (average 43 ±24 μm^2^, *p*<0.0001) which is unable to form mixed microcolonies (**Fig 8A** and **8C**, **S6 Movie**). As increasing microcolony size can result from the fusion of motile microcolonies [37, 38], we examined whether *N*. *cinerea* influences the motility of meningococci on cells. First, we measured the distance travelled by *N*. *meningitidis* microcolonies in the presence or absence of *N*. *cinerea* from 3–6 hpi. As shown in **Fig 8D**, the total distance migrated is significantly lower when *N*. *cinerea* wild-type or 346T*pilE*Δ1/2 is present (110 ±33 *vs*. 51 ±15 μm, *p*<0.0001, and 110 ±33 *vs*. 61 ±14 μm, *p* = 0.0004, respectively). In addition, in the presence of *N*. *cinerea*, a larger proportion of *N*. *meningitidis* microcolonies displayed almost no movement (*i*.*e*. <1 μm travelled between time points) compared to when meningococcus is present alone (S3 **Fig**, 41±5% of the total tracks compared with 21±11% respectively, *p* = 0.002). Similar results were obtained during coinfection with *N*. *cinerea pilE*Δ1/2 (S3 **Fig**, 21 ±11 *vs*. 35 ±10%, *p* = 0.04). Collectively, these data indicate that the presence of *N*. *cinerea* impairs *N*. *meningitidis* microcolony motility and expansion in a Tfp-independent manner.

## Discussion

While *N*. *meningitidis* can cause devastating systemic disease even in healthy children and adults, this pathogen more frequently displays a commensal type behaviour, asymptomatically colonising the human nasopharynx without progression to invasive disease [40]. As this niche is shared with commensal *Neisseria* species, there is potential for host-microbe and microbe-microbe interactions that may affect colonisation by one or other species. In this work we have characterised the interaction of the human commensal *N*. *cinerea* with respiratory epithelial cells and assessed the impact of this species on the cell-association of the closely-related, pathogenic *N*. *meningitidis*. Our data provide evidence of bacterial interference between *Neisseria* species.

Upon binding to epithelial cells, *N*. *meningitidis* induces the formation of specialised structures called cortical plaques which are enriched with actin, ezrin and other ERM proteins [6, 10, 41]. Our work reveals that several key cytoskeletal components recruited to cortical plaques also form honeycomb-like structures upon adhesion of *N*. *cinerea*. Interestingly, the accumulation of these proteins was not as pronounced as has been observed for the meningococcus [8, 10, 29, 42, 43]. This may reflect different experimental parameters, but may also be a consequence of quantitative or qualitative differences in host cell signalling induced by the different species. In agreement with the latter hypothesis, colocalisation of *N*. *cinerea* with CD44 occurs even in the absence of Tfp, whereas the pathogenic *Neisseria* require Tfp to modify the cortical cytoskeleton [6, 29]. For example retractile forces mediated by PilT contribute to formation and maintenance of cortical plaques by *N*. *gonorrhoeae* [38] and variant meningococcal pilin subunits recruit ezrin to different extents [29]. Thus, while the pathogen and commensal appear to co-opt the same molecules during adhesion, this may occur *via* different mechanisms.

Pathogenic *Neisseria* can form motile microcolonies on cell surfaces [37, 38]. Similarly we found that *N*. *cinerea* forms microcolonies which are Tfp-dependent, motile and undergo fusion, resulting in the formation of large, spherical aggregates. While it is well known that Tfp are involved in microcolony formation in pathogenic *Neisseria*, Tfp of *N*. *cinerea* do not have equivalent functions to meningococcal Tfp as they are unnecessary for adhesion [28]. Our findings thus reveal that pilus-mediated microcolony formation and motility are key features of the epicellular lifestyle of pathogenic and commensal *Neisseria*. Interestingly, we found no evidence of *N*. *cinerea* microcolony dispersal over the course of infection (16 h), suggesting that this may be a characteristic limited to pathogenic *Neisseria*. The biological significance of this is unclear, but dispersal may allow pathogenic *Neisseria* to disseminate, invade the epithelial layer and cause disease [13, 37, 44, 45]. Further work with other non-pathogenic *Neisseria* species is warranted, as defining pathogen specific properties linked to dissemination may provide insights into novel approaches for infection control.

Given the similarities between *N*. *cinerea* and *N*. *meningitidis* and their shared cell surface behaviour, we hypothesised that coinfection may influence the efficacy of cell association by one or the other species. Our data show that the presence of *N*. *cinerea* led to a decrease in the number of cell-associated meningococci. Interestingly this effect was observed both when *N*. *cinerea* was present on cells prior to infection with *N*. *meningitidis*, and when the two species were inoculated simultaneously. Analysis of co-infected cells using live cell microscopy established that this decrease correlates with reduced meningococcal microcolony size, although whether this is indeed the underlying cause remains to be determined. Microcolony expansion can result from the growth and division of bacteria or from the fusion of microcolonies [37, 38, 46]. Consistent with the latter, we observed a decrease in meningococcal microcolony movement in the presence of *N*. *cinerea*, which manifested as both a decrease in cumulative distance travelled and a larger proportion of microcolonies that moved only very small distances. This raises the question as to how the presence of *N*. *cinerea* could impact the movement of meningococcal microcolonies. Previous investigations have demonstrated that Tfp are central to microcolony movement and fusion [37]. Somewhat surprisingly, the reduced motility of *N*. *meningitidis* aggregates was also observed in the presence of non-piliated *N*. *cinerea* which suggests that it cannot simply be explained by *N*. *cinerea* anchoring and restraining meningococci *via* pilus-pilus interactions. Interestingly however, non-piliated *N*. *cinerea* still associates with components of cortical plaques, raising the possibility that the reduced motility arises because the commensal bacteria sequester cell components that would otherwise accumulate in high concentrations and co-migrate with motile meningococcal microcolonies [38]. Importantly, preventing aggregate fusion would result in smaller microcolonies, but not necessarily translate to an overall decrease in total bacteria associated with cells. Therefore, our data suggest that *N*. *cinerea* has multiple effects and may also influence meningococcal growth and/or interaction with cells. Possible explanations include occupying binding sites, inducing metabolic changes that impair meningococcal growth or cross-talk with host cells, or direct antagonism between the species on the cell surface.

A notable observation from our work is that there is clear evidence of segregation of the species within microcolonies and the two do not merge into spherical, well-mixed aggregates. Based on studies of *N*. *gonorrhoeae*, bacterial sorting in microcolonies is governed by Tfp and cells segregate if they have lowered pilus density or increased pilin glycosylation [39, 46]. As *N*. *cinerea* differs from meningococcus in pilin subunit sequence and Tfp function [28, 47] such bacterial sorting is not entirely unexpected, and our observations are consistent with lower breakage between pili of the different species [39].

As our knowledge of the composition and roles of the human microbiome increases, there is a corresponding need to understand the biology of commensal organisms and how they interact with both the human host and other bacteria at mucosal surfaces. Our work provides novel understanding of host-microbe interactions. We show that *N*. *cinerea* on human epithelial cells mimics some of the behaviour of *N*. *meningitidis*, but have identified subtle differences that distinguish the two species. Importantly we demonstrate that the two species physically interact on the cell surface and that *N*. *cinerea* impairs meningococcal association with cells. Our work suggests that commensal *Neisseria* residing in the nasopharynx could affect *N*. *meningitidis via* mechanisms that are independent of host immune responses and provides a new example of bacterial interference by commensal *Neisseria* species against their disease-causing relatives [21, 48].

## Materials and methods

### Bacterial strains and growth

Bacterial strains used in this study are shown in S1 **Table**. *Neisseria* spp. were grown on Brain Heart Infusion (BHI, Oxoid) agar with 5% defibrinated horse blood or in BHI liquid, at 37°C with 5% CO_2_. *E*. *coli* was grown on Luria Bertani (LB) agar or in liquid LB at 37°C with aeration. Antibiotics were added at the following concentrations: for *E*. *coli*, carbenicillin (carb) 100 μg/ml, and kanamycin (kan) 50 μg/ml; for *Neisseria* spp. kan 75 μg/ml, spectinomycin (spec) 65 μg/ml, erythromycin (ery) 15 μg/ml, and polymyxin B (pmB) 10 μg/ml.

### Antibodies and chemicals

Antibodies were used at the following final concentrations: α-CD44, 1:100 (clone F10-44-2, Abcam); α-Ezrin 1:250 (ab41672, Abcam). Phalloidin-Alexa Fluor 647 (Molecular Probes) was used to stain actin. Methyl-β-cyclodextrin (MβCD, Sigma-Aldrich) and water-soluble cholesterol (Sigma-Aldrich) were used at 5 mM and 130 μM, respectively. Filipin was dissolved in DMSO and used at final concentrations of 25 μg/ml. MβCD was present throughout experiments in the culture medium. The final concentration of DMSO did not exceed 0.1% and the impact of inhibitors at working concentrations on target and cell and bacterial viability was verified by microscopy, trypan blue exclusion assay and enumerating CFU respectively (S4 **Fig**).

### Strain construction

Primers used in this study are shown in S1 **Table**. pNCC101-sfCherry was generated in two steps. First, a fragment containing the *ori* and kanamycin resistance cassette of pUA139 [49] was amplified with primers pGL657/pGL658. Next, a fragment comprising the region from NEIS0479 to NEIS0482 in pNCC1 [28] was amplified with pGL91/pGL659. The two fragments were joined by Gibson Assembly (New England Biolabs), producing pNCC101. A codon-optimised gene encoding sfCherry [50] was synthesised (Thermo Fisher Scientific) and amplified using primers pGL599/pGL660, introducing *Xba*I and *Pac*I sites. The product was digested with these enzymes then ligated into pNCC101, generating pNCC101-sfCherry. The plasmid was linearized and used to transform *N*. *cinerea* 346T and *N*. *meningitidis* 8013, generating 346T_pNCC101sfCherry and 8013_pNCC101sfCherry, respectively.

### Analysis of cell association by live recovery

A549 human bronchial epithelial cells were cultured in Dulbecco’s modified Eagle’s medium (DMEM; Sigma) supplemented with 10% foetal bovine serum (FBS; Gibco) at 37°C in 5% CO_2_. Cells were seeded into 24-well plates at 2.5 x 10^5^ cells/well, and incubated overnight. Prior to infection, cells were washed three times with DMEM. Bacteria were grown overnight on solid media, resuspended in PBS and quantified using A260nm measurements [28]. The concentration of bacteria was adjusted to the desired level in culture media (± inhibitors) and the number of bacteria in the inocula was verified by plating dilutions to solid media. Cells were infected with 1 mL of bacterial suspension and incubated at 37°C in the presence of 5% CO_2_. Adhesion assays were carried out as previously [28] using an MOI of 30. Adhesion levels were calculated as the number of bacteria recovered from cells (output) / number of bacteria in the inoculum (input) expressed as a percentage.

For coinfection experiments, either *N*. *cinerea* was added to cells at an MOI of 50 and cells incubated for 4.5 h at 37°C and 5% CO_2_ then without further washing *N*. *meningitidis* was added at an MOI of 50 and cells incubated for a further 1.5 h. Alternatively, epithelial cells were simultaneously infected with *N*. *cinerea* or *E*. *coli* and *N*. *meningitidis* individually or in a 1:1 ratio, at a MOI of 50 for each and incubated for indicated times. Cells were then washed four times with PBS and cell-associated bacteria were recovered by lysing cells with 1mL of 1% saponin (MP Biomedicals) in PBS, mixing by repeat pipetting, and plating dilutions onto selective media (polymixin B for *N*. *meningitidis*, erythromycin for *N*. *cinerea*, carbenicillin for *E*. *coli*). The total number of bacteria recovered from the well (entire monolayer) was calculated and expressed as CFU/Well.

### Confocal microscopy

Epithelial cells were seeded at a density of 10^5^ cells/ml onto glass coverslips and infected with bacteria at MOI of 100. At various times post infection cells were washed three times with PBS, fixed with paraformaldehyde (4% w/v in PBS for 20 min), then permeabilised with saponin (0.5% w/v in PBS for 10 min). Coverslips were incubated with primary antibodies overnight at 4°C in 1% (w/v) bovine serum albumin (BSA) in PBS, washed three times with PBS, and incubated for 1 h with either a goat anti-rabbit pAb, or an anti-mouse IgG-Alexa 647 conjugated pAb (Molecular probes). Actin or cholesterol staining were performed for 1 h at room temperature. Cells were then washed three times with PBS, mounted onto slides using Vectashield (Vector Laboratories) with diamidino-2-phenylindole (DAPI), and visualized using an Olympus Fluoview FV1200 equipped with an Olympus UPLanSApo 100x/1.40 objective. Serial Z-stacks were taken with 0.2–0.3 μm slices. For quantification, the frequency of protein co-localisation underneath bacteria or microcolonies was determined by counting at least 50 events unless otherwise stated. A microcolony was defined as a cluster of ≥5 bacteria. Co-localisation was scored when a honeycomb lattice arrangement was detected [51]. Image analysis and processing was performed using Fiji [52].

### Scanning Electron Microscopy (SEM)

For SEM A549 cells were seeded as described above and infected for 6 h at an MOI of 100 with *N*. *cinerea* and *N*. *meningitidis* individually or in a 1:1 ratio. Infected cells were washed three times with pre-warmed PBS and fixed for 20 min with 1 ml of PBS containing 0.5% glutaraldehyde (AppliChem) and 2% paraformaldehyde (Sigma). Coverslips were washed as above, then stained with 1% OsO_4_, 0.1M PIPES buffer for 1 h at 4°C, washed three times with deionized water and taken through an ethanol dehydration series (50, 70, 90, 95% ethanol for 5 min each, then 100% ethanol three times for 15 min). Coverslips were dried with hexamethyldisilazane for 3 min, then mounted on carbon adhesive tape on an SEM stub and sputter coated with ~15 nm layer of gold. Images were acquired using a Zeiss Sigma 300 Field Emission Gun SEM operated at 2.0 kV.

### Live-cell imaging and image analysis

For live-cell imaging we used an EVOS FL Auto Imaging System (Life Technologies) equipped with GFP (470/22 Ex; 510/42 Em) and Texas Red (585/29 Ex; 624/40 Em) LED cubes. The microscope stage was maintained at 37°C and 5% CO_2_.

To analyse dynamics of *N*. *cinerea* on epithelial cells A549 cells were seeded at 2.5 x 10^5^ cells/well into 24-well plates, and bacteria added at an MOI of 50 for 1.5 h. After three washes, fresh media was added to cells and images acquired at 10 min intervals over 16 hours. For imaging of *N*. *meningitidis* in presence or absence of *N*. *cinerea*, A549 cells were seeded as above and infected with either *N*. *meningitidis* alone or *N*. *meningitidis* and *N*. *cinerea*, each at an MOI of 50. Live imaging commenced after 1.5 h of infection (without washing) and images were acquired at 10 min intervals over the subsequent 4.5 h using Invitrogen EVOS FL Auto software. Processing was performed using Fiji, briefly, individual images of each fluorescent channel were imported sequentially using Image Sequence and merged to produce AVI. Files.

### Microcolony analysis

A microcolony was defined as a persistent bacterial aggregate with a diameter > 5 μm. At indicated timepoints a Region of Interest (ROI) was manually drawn around each microcolony and using the “measure” tool in Fiji the area of the bacterial aggregate was calculated. To measure microcolony movement, each microcolony was tracked individually over time using the “manual tracking” tool, which identifies the *x* and *y* coordinates of individual microcolonies in each frame of a time lapse sequence. At each timepoint, the centre of the microcolony was mapped and the distance travelled calculated by comparison to its coordinate at the previous timepoint. For these analyses we excluded microcolonies that moved out of the field of view. The size and displacement of the microcolonies at sequential time points were analysed in Graphpad Prism7 software. In total, we analysed four microcolonies per image, from 19 sequential frames acquired from one well for each condition (*i*.*e*. single infection and co infection). This resulted in a total of 648 different measurements per condition, from nine replicates and three independent experiments.

### Statistical analyses

Graphpad Prism7 software (San Diego, CA) was used for statistical analysis. We used One-way/two-way ANOVA with Tukey post hoc testing for multiple comparisons and Unpaired two-tailed Student’s t-test for pairwise comparisons. In all cases, *p* <0.05 was considered statistically significant.

## Supporting information

S1 Fig **(A) *N*. *meningitidis*-induced recruitment of cortical plaque components in A549 epithelial cells.** A549 cells infected with *N*. *meningitidis* 8013 at MOI 100 for 3 h were immunostained for actin, ezrin or CD44 and analysed by microscopy (see Materials and methods). Bacterial DNA and epithelial cell nuclei were stained with DAPI (blue), cortical plaque proteins actin, CD44 and ezrin are shown in red (merge panels). White arrows highlight recruitment of proteins to attachment site of microcolonies. Scale bars correspond to 10 μm. **(B) Cholesterol is not recruited to the site of *N*. *cinerea* attachment.** Infected cells with *N*. *cinerea* wild-type (wt) expressing sfGFP were fixed at 3 hpi and host plasma-membrane cholesterol was detected with filipin (incubation with 25 μg/ml for 1 h at room temperature). Magnified area in the panel on the right highlights cholesterol distribution in an infected cell. No visible enrichment of cholesterol was observed underneath *N*. *cinerea* microcolonies. Scale bar corresponds to 10 μm.
(TIF)

S2 Fig *N. cinerea* ACP homologue contributes to epithelial cell adhesion. (**A**) The *N*. *cinerea* homologue of *acp* was translated and amino acid sequence was aligned with ACP from *N*. *meningitidis* 8013 using Clustal Omega. Percent identity was also calculated using Clustal Omega [54]. (**B**) A549 cells were infected for 0.5, 1.5 and 3 h either with wild-type *N*. *cinerea* 346T (Wt) or 346TΔ*NEIS2075* (Δ*acp*) at MOI of 30. Adhesion levels were quantified by enumeration of cell-associated bacteria. Data shown represent the mean +SD of three independent experiments carried out in triplicate. NS, not significant; *p<0.05; (unpaired two-tailed Student’s t-test).
(TIF)

S3 Fig *N. cinerea* reduces the movement of *N. meningitidis* microcolonies. Movement of meningococcal (*Nm*8013) microcolonies on A549 epithelial cells. (**A**-**C**) Distance travelled by each microcolony over 10 min intervals, when alone (**A**), or during coinfection with wild-type *N*. *cinerea* (**B**) or *N*. *cinerea* Δ*pilE*1/2 (**C**). Each line corresponds to a single microcolony tracked between 3 and 6 hpi. Data are from a total of 36 microcolonies from three independent experiments. (**D**) Percentage of microcolonies moving different distances (indicated) over each 10 min interval in presence or absence of *N*. *cinerea*. Data shown represent the mean +SD of three independent experiments performed in triplicate. NS, not significant; *, *p*<0.05; **, *p*< 0.005 (two-way ANOVA test).
(TIF)

S4 Fig Impact of MβCD cholesterol depleting agent on A549 cell viability and *N. cinerea* survival. **(A)** A549 cells were treated with MβCD (5 mM), MβCD+Chol (5 mM and 130 μM, respectively) for 3 h in complete tissue culture media. Cell viability was calculated as the number of viable cells divided by the total number of cells within the grids on a haemocytometer. Cells stained with trypan blue were considered non-viable. **(B)** *N*. *cinerea* 346T was incubated with drugs as above or left untreated (NT) in DMEM with 10% FBS. After 3 h, bacterial numbers were determined by serial dilution and plating. No difference in bacterial viability (CFU/mL) was found compared to non-treated control. Data shown represent the mean +SD of two independent experiments carried out in triplicate. NS, Not significant.
(TIF)

S1 Table List of bacterial strains and primers used in this study. (DOCX)

S1 Movie Time lapse images of A549 epithelial cells infected with *N. cinerea* wild-type (Wt) expressing sfCherry at an MOI of 50 for 16 h. Images were captured at 10 min intervals and each frame of the movie corresponds to a 10 min interval. Time lapse video representative of three independent experiments performed in triplicate. Still images of the movie are available in Fig 4A.
(AVI)

S2 Movie Time lapse images of A549 epithelial cells infected with *N. cinerea* 346TΔ*pilE1/2* expressing GFP at an MOI of 50 for 16 h. Images were captured at 10 min intervals and each frame of the movie corresponds to a 10 min interval. Time lapse video representative of three independent experiments performed in triplicate. Non-piliated *N*. *cinerea* failed to form visible microcolonies over time. Still images of the movie are available in Fig 4C.
(AVI)

S3 Movie Time lapse images of A549 epithelial cells co-infected with *N. cinerea* wild-type (Wt) expressing sfCherry and *N. cinerea* 346TΔ*pilE1/2* expressing GFP both at an MOI of 50 for 16 h. Images were captured at 10 min intervals and each frame of the movie corresponds to a 10 min interval. Time lapse video representative of three independent experiments performed in triplicate. Still images of the movie are available in Fig 4D.
(AVI)

S4 Movie Time lapse images of A549 epithelial cells infected with *N. meningitidis* 8013 wild-type (Wt) expressing sfCherry at an MOI of 50 for 6 h. Images were captured at 10 min intervals and each frame of the movie corresponds to a 10 min interval. The movie covers a 6 h period from 1.5 h post infection. *N*. *meningitidis* forms motile microcolonies which enlarged throughout infection. Time lapse video representative of three independent experiments performed in triplicate. Still images of the movie are available in Fig 8A.
(AVI)

S5 Movie Time lapse images of A549 epithelial cells co-infected with *N. meningitidis* 8013 wild-type (Wt) expressing sfCherry and *N. cinerea* wild-type (Wt) expressing GFP both at an MOI of 50 for 6 h. Images were captured at 10 min intervals and each frame of the movie corresponds to a 10 min interval. The movie covers a 6 h period from 1.5 h post infection. *N*. *cinerea* and *N*. *meningitidis* form mixed multi-lobed microcolonies and the expansion of meningococcal microcolonies is visibly reduced by the presence of commensal *Neisseria*. Time lapse video representative of three independent experiments performed in triplicate. Still images of the movie are available in Fig 8A.
(AVI)

S6 Movie Time lapse images of A549 epithelial cells co-infected with *N. meningitidis* 8013 wild-type (Wt) expressing sfCherry and *N. cinerea* 346TΔ*pilE1/2* expressing GFP both at an MOI of 50 for 6 h. Images were captured at 10 min intervals and each frame of the movie corresponds to a 10 min interval. The movie covers a 6 h period from 1.5 h post infection. Non-piliated *N*. *cinerea* and meningococcus do not form mixed microcolonies. Time lapse video representative of three independent experiments performed in triplicate. Still images of the movie are available in Fig 8A.
(AVI)
