# Supplementary figures and images for: Commensal Neisseria cinerea impairs Neisseria meningitidis microcolony development and reduces pathogen colonisation of epithelial cells
Source: PLoS Pathog. 2020 Mar 24;16(3):e1008372. doi: 10.1371/journal.ppat.1008372 (PMC7092958; doi:10.1371/journal.ppat.1008372)

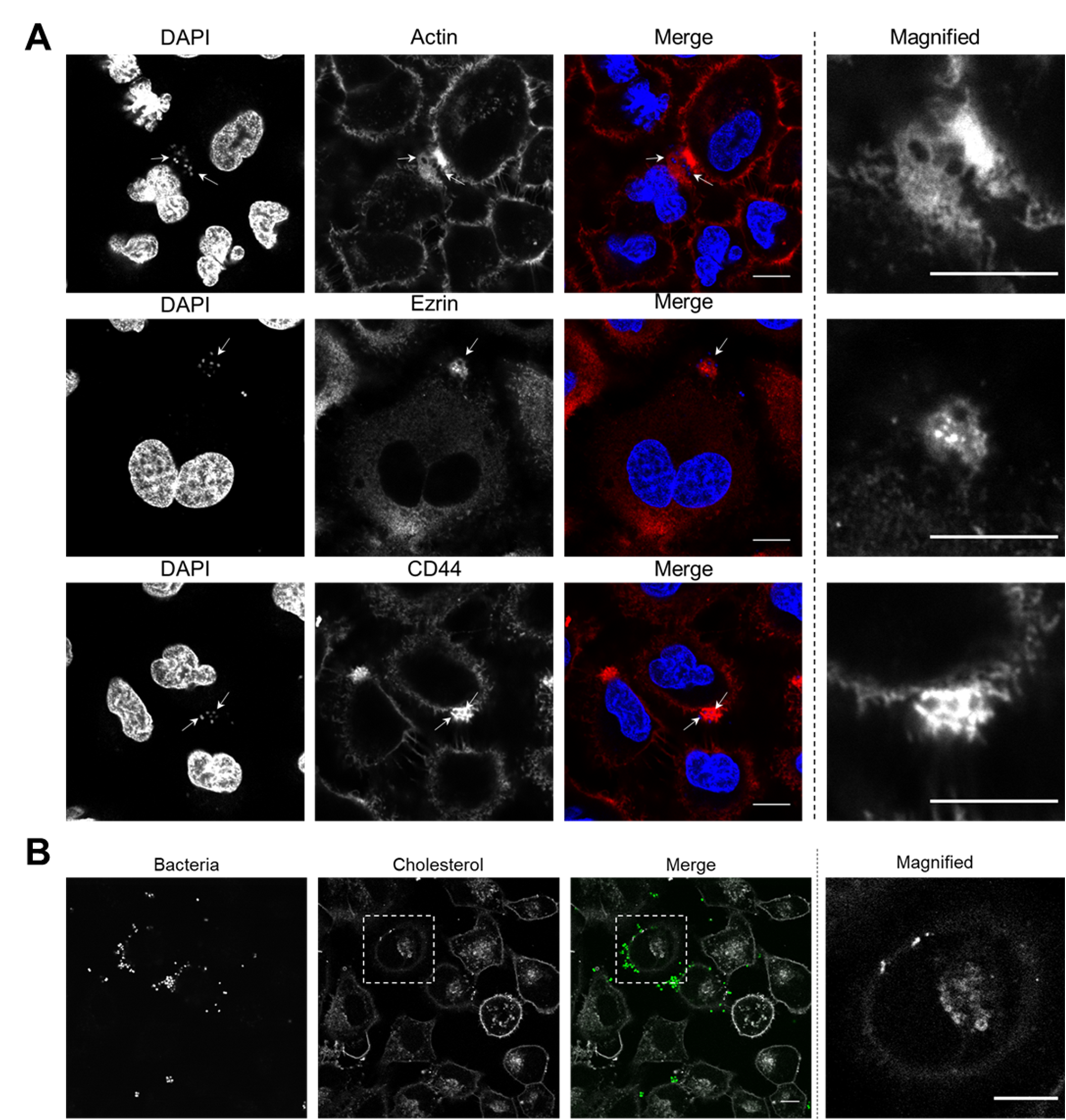

Supplement: S1 Fig — (A) N. meningitidis-induced recruitment of cortical plaque components in A549 epithelial cells. A549 cells infected with N. meningitidis 8013 at MOI 100 for 3 h were immunostained for actin, ezrin or CD44 and analysed by microscopy (see Materials and methods). Bacterial DNA and epithelial cell nuclei were stained with DAPI (blue), cortical plaque proteins actin, CD44 and ezrin are shown in red (merge panels). White arrows highlight recruitment of proteins to attachment site of microcolonies. Scale bars correspond to 10 μm. (B) Cholesterol is not recruited to the site of N. cinerea attachment. Infected cells with N. cinerea wild-type (wt) expressing sfGFP were fixed at 3 hpi and host plasma-membrane cholesterol was detected with filipin (incubation with 25 μg/ml for 1 h at room temperature). Magnified area in the panel on the right highlights cholesterol distribution in an infected cell. No visible enrichment of cholesterol was observed underneath N. cinerea microcolonies. Scale bar corresponds to 10 μm. (TIF) [file ppat.1008372.s001.tif]

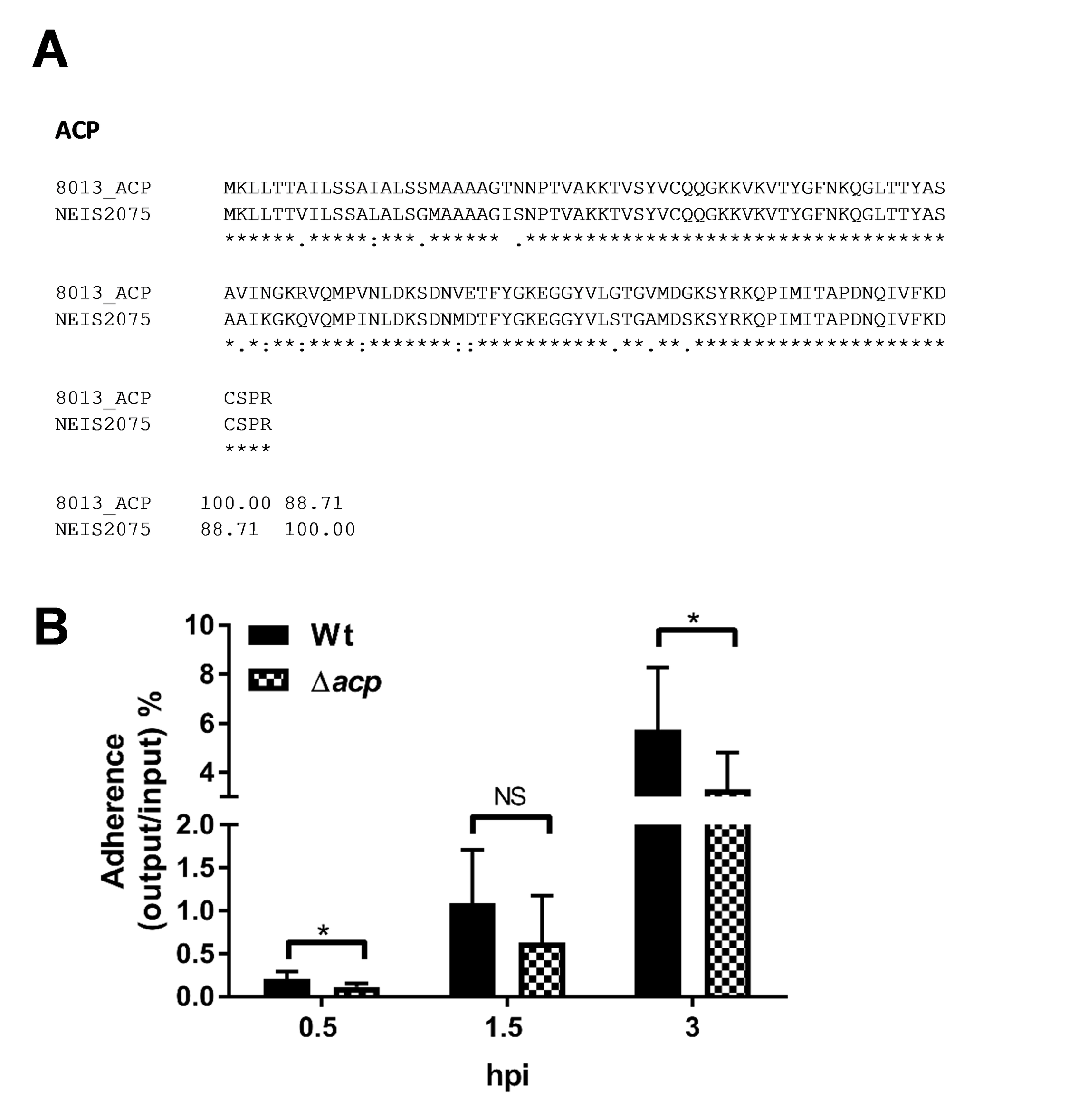

Supplement: S2 Fig — (A) The N. cinerea homologue of acp was translated and amino acid sequence was aligned with ACP from N. meningitidis 8013 using Clustal Omega. Percent identity was also calculated using Clustal Omega [54]. (B) A549 cells were infected for 0.5, 1.5 and 3 h either with wild-type N. cinerea 346T (Wt) or 346TΔNEIS2075 (Δacp) at MOI of 30. Adhesion levels were quantified by enumeration of cell-associated bacteria. Data shown represent the mean +SD of three independent experiments carried out in triplicate. NS, not significant; *p<0.05; (unpaired two-tailed Student’s t-test). (TIF) [file ppat.1008372.s002.tif]

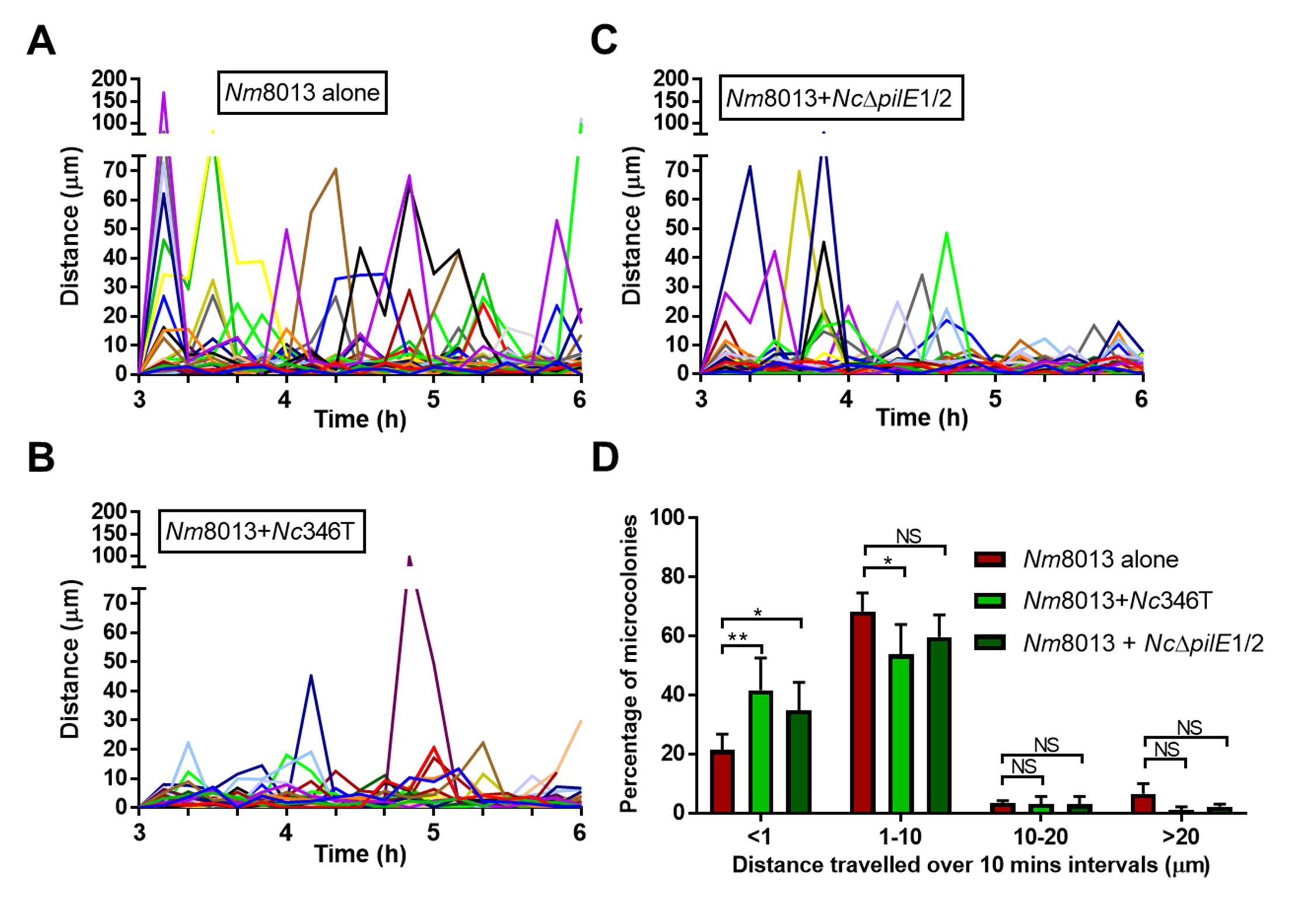

Supplement: S3 Fig — Movement of meningococcal (Nm8013) microcolonies on A549 epithelial cells. (A-C) Distance travelled by each microcolony over 10 min intervals, when alone (A), or during coinfection with wild-type N. cinerea (B) or N. cinerea ΔpilE1/2 (C). Each line corresponds to a single microcolony tracked between 3 and 6 hpi. Data are from a total of 36 microcolonies from three independent experiments. (D) Percentage of microcolonies moving different distances (indicated) over each 10 min interval in presence or absence of N. cinerea. Data shown represent the mean +SD of three independent experiments performed in triplicate. NS, not significant; *, p<0.05; **, p< 0.005 (two-way ANOVA test). (TIF) [file ppat.1008372.s003.tif]

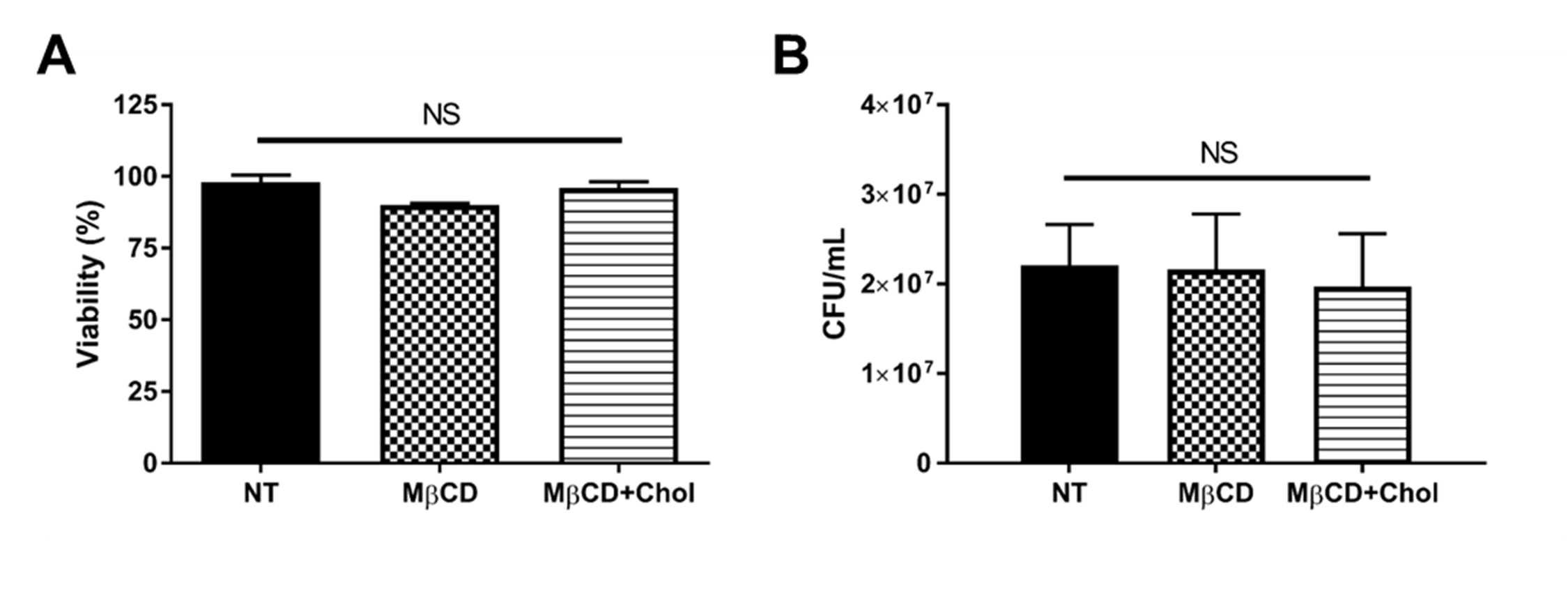

Supplement: S4 Fig — (A) A549 cells were treated with MβCD (5 mM), MβCD+Chol (5 mM and 130 μM, respectively) for 3 h in complete tissue culture media. Cell viability was calculated as the number of viable cells divided by the total number of cells within the grids on a haemocytometer. Cells stained with trypan blue were considered non-viable. (B) N. cinerea 346T was incubated with drugs as above or left untreated (NT) in DMEM with 10% FBS. After 3 h, bacterial numbers were determined by serial dilution and plating. No difference in bacterial viability (CFU/mL) was found compared to non-treated control. Data shown represent the mean +SD of two independent experiments carried out in triplicate. NS, Not significant. (TIF) [file ppat.1008372.s004.tif]
