# Supplementary material for: Commensal Neisseria cinerea impairs Neisseria meningitidis microcolony development and reduces pathogen colonisation of epithelial cells
Source: PLoS Pathog. 2020 Mar 24;16(3):e1008372. doi: 10.1371/journal.ppat.1008372 (PMC7092958; doi:10.1371/journal.ppat.1008372)
Supplement: S1 Table — (DOCX) [file ppat.1008372.s005.docx]

**S1 Table**. Bacterial strains and primers used in this study.

| **Strains /Primer name** | **Description / sequence** | **Reference/ Source** |
| --- | --- | --- |
| ***Neisseria cinerea*** |  |  |
| CCUG346T (346T) | *N. cinerea* wild-type | (1) |
| 346T_pNCC1sfGFP (WTsfGFP) | sfGFP-expressing 346T; Ery^R^ | (2) |
| 346TΔ*pilE*1/2_pNCC1sfGFP (Δ*pilE*1/2sfGFP) | sfGFP-expressing 346TΔ*pilE*1/2;Kan^R^ Ery^R^ | (2) |
| 346TΔ*NEIS2075* | deletion mutagenesis, NEIS2075 deficient; Ery^R^ | This study |
| 346T_pNCC101sfCherry (WTsfCherry) | sfCherry-expressing 346T; Ery | This study |
| ***Neisseria meningitidis*** |  |  |
| 8013 | Wild-type Serogroup C *N. meningitidis* | (3) |
| 8013_pNCC101sfCherry (WTsfCherry) | sfCherry-expressing 8013; Ery^R^ | This study |
| ***Escherichia coli*** |  |  |
| Dh5α | Cloning strain | Lab collection |
| BL21 pET21b | Amp^R^ | Lab collection |
| Dh5αpGEM-TEZΔ*NEIS2075* | NEIS2075 deletion construct; Carb^R^ Ery^R^ | This study |
| ***Primers*** |  |  |
| pGL91 | CCAACCTGCCATCACGAGATTTCGATTCCACCGCCGCCTTTTACAGTACTTTTACGATGCTTTC | This study |
| pGL599 | CCCTCTAGATTTTAACCACCGGTACTATGACGAC | This study |
| pGL657 | ATCTCGTGATGGCAGGTTGGCTATTTCTTCCAGAATTGCCATG | This study |
| pGL658 | AATCATCAGTTGGGCTACAGGATTGGGACTCGGAATTGCCAGCTGGG | This study |
| pGL659 | AGTCCCAATCCTGTAGCCCA | This study |
| pGL660 | CCCTTAATTAATTGACAGCTAGCTCAGTCCTAGGTATAATGCTAGCCCAACATGTTACACAATAATGGAGTAATGAACATATGGAAGAAGATAACATGGCC | This study |

**REFERENCES**

1. Bennett JS, Jolley KA, Earle SG, Corton C, Bentley SD, Parkhill J, Maiden MC. 2012. A genomic approach to bacterial taxonomy: an examination and proposed reclassification of species within the genus *Neisseria*. Microbiology 158:1570-80.

2. Wormann ME, Horien CL, Johnson E, Liu G, Aho E, Tang CM, Exley RM. 2016. *Neisseria cinerea* isolates can adhere to human epithelial cells by type IV pilus-independent mechanisms. Microbiology 162:487-502.

3. Rusniok C, Vallenet D, Floquet S, Ewles H, Mouze-Soulama C, Brown D, Lajus A, Buchrieser C, Medigue C, Glaser P, Pelicic V. 2009. NeMeSys: a biological resource for narrowing the gap between sequence and function in the human pathogen *Neisseria meningitidis*. Genome Biol 10:R110.
